# A Novel *ARMC5* Germline Variant in Primary Macronodular Adrenal Hyperplasia Using Whole-Exome Sequencing

**DOI:** 10.3390/diagnostics12123028

**Published:** 2022-12-02

**Authors:** Maryam Eghbali, Sara Cheraghi, Sara Samanian, Iman Rad, Jafar Meghdadi, Hamideh Akbari, Maryam Honardoost

**Affiliations:** 1 Endocrine Research Center, Institute of Endocrinology and Metabolism, Iran University of Medical Sciences, Tehran 1593716615, Iran; 2 Genetic Foundation of Tehran, Solaleh Diagnostic Laboratory, Tehran 1393845944, Iran; 3 Stem Cell Technology Research Center (STRC), Tehran 1665666311, Iran; 4 Clinical Research Development Unit (CRDU), Sayad Shirazi Hospital, Golestan University of Medical Sciences, Gorgan 49178677439, Iran

**Keywords:** PMAH, whole-exome sequencing, *ARMC5*, novel germline variant

## Abstract

Background: Primary macronodular adrenocortical hyperplasia (PMAH) is a rare form of adrenal Cushing’s syndrome with incomplete penetrance which may be sporadic or autosomal dominant. The inactivation of the *ARMC5* gene, a potential tumor suppressor gene, is one of the associated causes of PMAH. This study aimed to identify the variant responsible for Iranian familial PMAH. Methods: The proband, a 44-year-old woman, was directed to whole-exome sequencing (WES) of the blood sample to discover a germline variant. In addition, the identified causative variant was confirmed and segregated in other and available unaffected family members. Results: The novel germline heterozygous missense variant, c.2105C>A in the *ARMC5* gene, was found, and the same germline variant as the proband was confirmed in two affected sisters. This variant was detected in the brother of the proband with an asymptomatic condition and this considered because of incomplete penetrance and age-dependent appearance. The function of the *ARMC5* protein would be damaged by the identified variant, according to in silico and computer analyses that followed. Conclusion: The new germline *ARMC5* variation (c.2105C>A, (p. Ala702Glu)) was interpreted as a likely pathogenic variant based on ACMG and Sherloc standards. PMAH may be diagnosed early using genetic testing that shows inherited autosomal dominant mutations in the *ARMC5* gene.

## 1. Introduction 

Primary macronodular adrenal hyperplasia (PMAH) is an endogenous Cushing’s syndrome (CS) that represents < 2% of CS cases [1,2,3,4]. It is characteristically diagnosed during the 1950s to 1960s and is mostly associated with mild hypercortisolism and multiple nodules in the adrenal glands [1]. PMAH was initially reported as a sporadic disease but is found to be dominantly inherited in several families [5]. 

**Figure 1:**
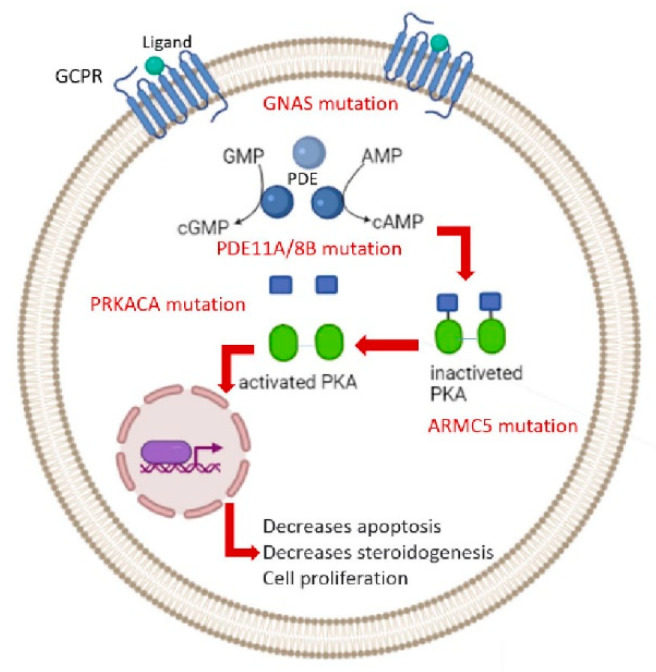
Genetics of Primary macronodular adrenal hyperplasia.

Numerous molecular mechanisms have contributed to the pathogenesis of PMAH, mostly the cyclic AMP/protein kinase A (cAMP/PKA) signaling pathway (Figure 1) [1]. Using an integrated genomics approach in 2013, the loss of heterozygosity (LOH) on the short arm of chromosome 16 has been identiﬁed as the first responsible genetic defect for AIMAH [6]. Subsequently, using a single-nucleotide polymorphism (SNP) array, frequent events in adrenal tumor tissues have been detected [4]. Recent advances in molecular genetics research revealed germline and somatic mutations of several genes were involved in the mechanisms causing PMAH [7].

For instance, mutations in tumor suppressor genes, such as adenomatous polyposis coli gene (*APC*), menin (*MEN1*), fumarate hydratase (*FH*), and armadillo repeat-containing protein 5 (*ARMC5*), which later is detected in 20% to 50% of PMAH cases [8]. Recently, the other tumor suppressor gene, lysine (K)-specific demethylase 1A (*KDM1A)* was identified in PMAH associated with food-dependent Cushing’s syndrome [9,10,11,12]. Furthermore, mutations in the ACTH receptor gene and guanine nucleotide-binding protein alpha-stimulating activity polypeptide (*GNAS*), cAMP-dependent protein kinase catalytic subunit alpha (*PRKACA*), phosphodiesterase 11A (*PDE11A*), and phosphodiesterase 8B (*PDE8B*) less frequently occurred in patients [8].

*ARMC5* germline mutations are responsible for up to 50% (near to 80% in Japanese) of familial or sporadic PMAH and lead to larger adrenal hyperplasia, higher hypercortisolism, a higher number of nodules, hypertension, higher fasting glucose, and HbA1c [4,6]. Owing to the disease severity, patients carrying *ARMC5* mutations often need surgery [6]. Therefore, awareness of a patient’s *ARMC5* status will support the diagnosis of PMAH. Moreover, screening family members of affected patients may help clinicians in the early identification and prevention of morbidity and even mortality caused by CS. In this study, we performed clinical data collection and whole exome sequencing (WES) to discover the disease-causing variant in one PMAH family. A novel germline variant in the *ARMC5* gene was detected, and the candidate variant in the family members was confirmed and segregated by Sanger sequencing. 

## 2. Materials and Methods

### 2.1. Sample Collection and Ethics Statement

This research was approved by the ethics committee of the Iran University of Medical Sciences, Tehran, IRAN, and all study subjects signed the informed consent for genetic investigation and publication of clinical evidence. Available members of the pedigree were enrolled in this manuscript. Germline genomic DNAs from four patients (II-3, III-3, III-5, and III-6) with the clinical diagnosis of primary macronodular adrenocortical hyperplasia (PMAH) and four unaffected family members (III-2, III-7, III-8, and IV-2) were isolated from the blood samples by applying the standard salting-out standard protocol [13]. In addition, the available tumor genomic DNA of these patients (III-3, III-5, and III-6) was extracted using the RIBO-prep nucleic acid extraction kit (AmpliSens). 

Each patient experienced clinical, laboratory, and radiological assessments for the presence of PMAH. All medical records of healthcare, routine physical, and fundus examinations were collected. Whole exome sequencing was only performed on proband III-6 and recognized variants confirmed by Sanger sequencing in other mentioned family members.

### 2.2. Whole-Exome Sequencing and Bioinformatics Analysis

Whole-exome sequencing through the Illumina NextSeq platform was performed for proband III-6. In the library preparation step, a paired-end DNA sequencing library was prepared using the Agilent SureSelect V7 Target Enrichment Kit (Agilent Technologies, Santa Clara, CA, USA). The constructed library was sequenced with a mean coverage of nearly 200X. After the base calling and quality assessment of sequencing data, paired-end 150-bp sequence reads were mapped to the UCSC human reference genome (GRCh37/hg19 assembly) by BWA with ‘mem’ mode [14]. The following was performed by removing duplicates and low-quality reads (QBase < 20). 

After mapping, the generated Sequence Alignment/Map (SAM) file was converted to a BAM file using Samtools [15], and this software was also used for sorting and indexing the bam file. The created BAM and BAM index files were applied for the viewing variant’s location and visualization of the read depth by an integrated genome viewer (IGV) softwareversion 2.8.2 [16]. Variant calling of single nucleotide variants (SNVs) and short insertions or deletions (Indels) were performed through the GATK (v4.1.9.0) tool. The output of this step was a variant call format (VCF) file and subsequently was submitted to the wANNOVAR (the access date: 22 October 2021) (http://wannovar.wglab.org/ access date: 22 October 2021) and Franklin (https://franklin.genoox.com/ access date: 22 October 2021) for annotating.

**Figure 2:**
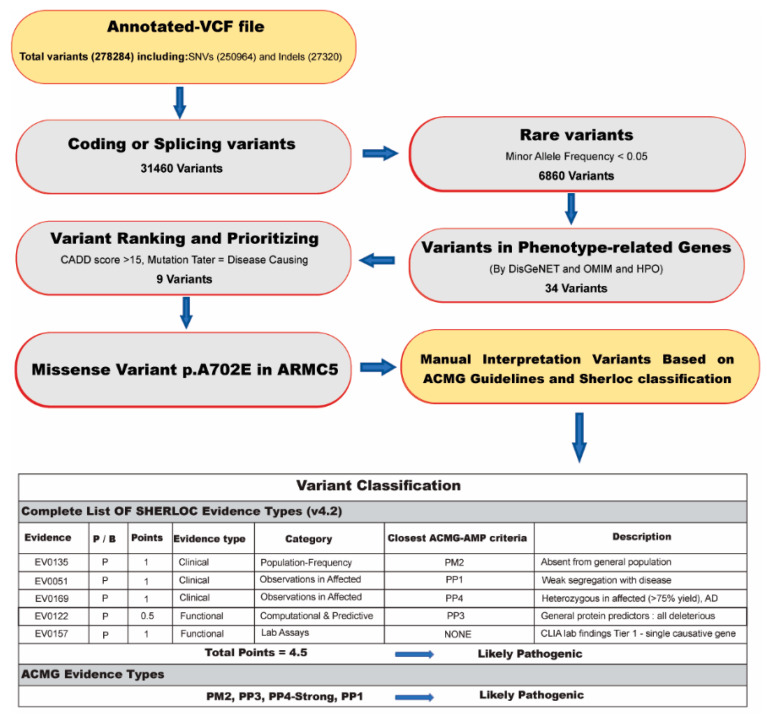
The workflow of determining the disease-causing variant for the PMAH family.

An in-house filtering pipeline was used to detect the proband’s candidate causative variant/variants. The stepwise approach for data analysis is mentioned in (Figure 2). In this regard, common variants were excluded with minor allele frequency exceeding 0.05 using datasets from dbSNP4, 1000 Genomes Project, ExAC database, ESP (Exon Sequencing Projects), gnomAD database, and Iranome (http://www.iranome.ir/ access date: 22 October 2021). Then, intronic, synonymous, upstream/downstream variants were removed, and only point variants and indels (<20 bp) located in exonic regions or canonical splicing sites were included in annotated files for further analysis. 

The residual variants were prioritized and filtered as follows:

(1) Including and selecting known/unknown missense variants and loss-of-function (LOF) variants.
(2) Checking variants in the Human Gene Mutation Database (HGMD) (http://www.hgmd.cf.ac.uk/ac/ access date: 22 October 2021) and ClinVar (https://www.ncbi.nlm.nih.gov/clinvar access date: 22 October 2021) to detect formerly reported mutations as pathogenic or likely pathogenic.
(3) In silico analysis was directed by PolyPhen2 [17], Combined Annotation Dependent Depletion (CADD) [18], Mutation Taster [19], DANN score [20], HOPE web (https://www3.cmbi.umcn.nl/hope/ access date: 22 October 2021), and GERP score [21] to evaluate the potential pathogenicity of the variants based on the function or structure prediction. Additionally, the GERP score was used to assess the conservation score.
(4) To find related variants with the patient’s clinical information, we used the Human Phenotype Ontology (HPO) as the phenotype–gene association database and the Online Mendelian Inheritance in Man (OMIM) as the gene–disease association database to discover the damaged genes related to the phenotype of the patients.
(5) Finally, the interpretation of the novel variants was completed using the manual approach to the American College of Medical Genetics and Genomics (ACMG) guidelines [22] and semiquantitative-, hierarchical evidence-based rules for locus interpretation (Sherloc) [23].

### 2.3. The Variant Validation and Co-Segregation Analysis

Following data filtering, the detected causative variant was confirmed by Sanger sequencing in DNA samples of the proband and other family members to show co-segregation of this variant in the causative genes related to the PMAH phenotype. For this purpose, specific primers were designed with the Primer3 website and Gene Runner 6.0 software for the region including the detected variant, and PCR-amplified products were sequenced by the ABI 3500 Genetic Analyzer (Applied Biosystems, Foster City, CA, US). For data analysis of the sequence, traces were used in the Ensemble reference sequences and Chromas v2.33 software, and CodonCode aligner software.

### 2.4. Protein Structure Analysis 

Original protein sequences of *ARMC5* were retrieved from UniProtKB with ID of Q96C12 (*ARMC5*_HUMAN), which was cross-referenced with other IDs such as NP_001275969, NM_001105247.2, and XP_005255637. Since no structure information was available for the area of interest (between ARM7 repeat and BTB homo-dimerization domain) in *ARMC5* protein using the SWISS-MODEL Repository (SMR), the Protein Homology/analogY Recognition Engine (Phyre v2.0) was used to predict the secondary structure of *ARMC5* [24,25]. Then, the predicted secondary structure of both *ARMC5* variants (either with Ala702 or Glu702 in the 445–747 region, which from now on will be called “*ARMC5*-Ala702” and “*ARMC5*-Glu702”) were compared. The tertiary structure of 1XQR with 97.9% confidence was chosen as the highest-scoring template. Substitution of His58 by either Ala or Glu residues in 1XQR[A]-5 was performed using the mutate tool of Hyperchem software (v 8.0.10) [26], which resulted in the development of 1XQR[A]-5-Ala58 and 1XQR[A]-5-Glu58 variants, respectively. Optimizing the geometry of the 1XQR[A]-5 (amino acids of 274–350) was performed using the Polak–Ribiere algorithm, as well as 1XQR[A]-5 variants. To calculate the hydrophobicity of *ARMC5* variants, their corresponding sequences were tested using the GRAVY index of ProtParam from Expacy online service [27].

## 3. Results

### 3.1. Subjects

**Figure 3:**
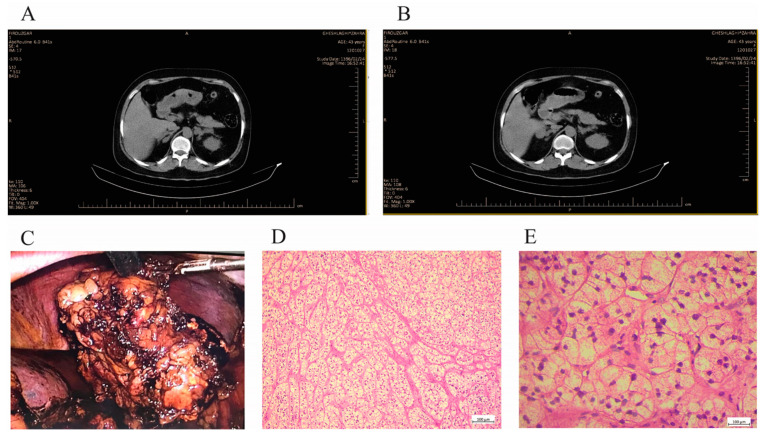
Clinical data of the proband (III-6). (**A**,**B**) Abdominal CT scan images showed bilateral irregular adrenal masses, adrenal glands are replaced by multiple nodules. (**C**) Macroscopic cross-sectional view of the resected formaldehyde fixed right adrenal gland with yellowish nodules of various sizes. (**D**,**E**) Histological view of hematoxylin-eosin (HE) staining of resected tissues showed diffuse hyperplasia of zona fasciculate.

**Figure 4:**
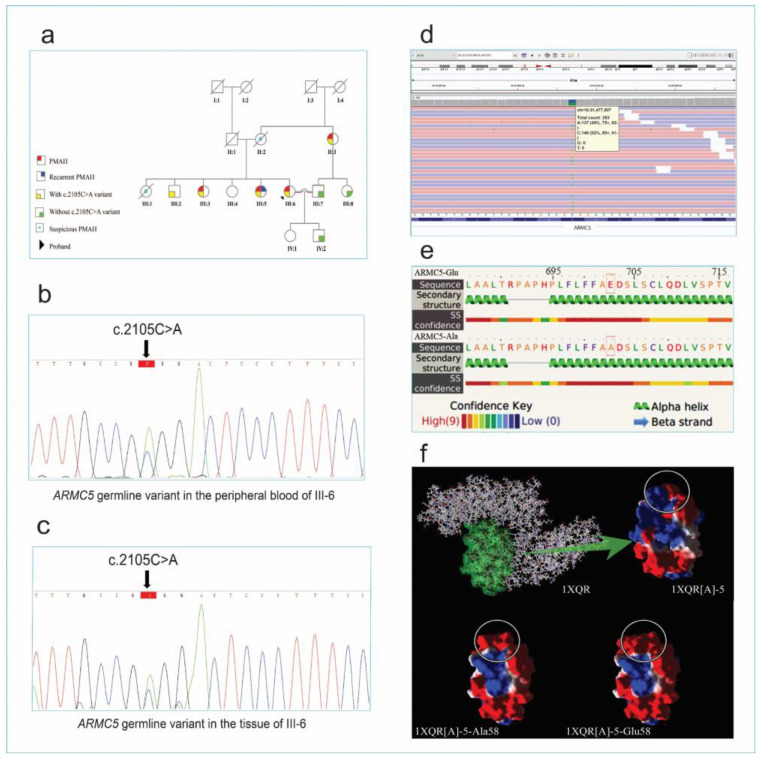
The pedigree of the family with familial PMAH, predicted secondary structure of *ARMC5* and its tertiary structure (Adopted from 1XQR), BAM interfaces, and sanger confirmation results. (**a**) The pedigree of the patient (III-6). The arrows indicates the proband. (**b**) Sanger sequencing confirmation for the candidate causative variant of *ARMC5* in the peripheral blood sample and (**c**) the tissue of the Patient (III-6). (**d**) The WES reads were visualized by using the Integrative Genomics Viewer (IGV). (**e**) Secondary structures of *ARMC5* variants were obtained, using the Phyre online service. The substituted residues are highlighted in orange rectangles. (**f**) Tertiary structure of 1XQR is demonstrated in the top left. The 1XQR[A]-5 domain is highlighted in green, which is also electrostatically colored (on the right side of the arrow). The red, white, and blue represent negative, neutral, and positive charges, respectively, and the white circle represents the position of residues of interest either in 1XQR[A]-5 or in its variants. In 1XQR[A]-5 variants’, His58 is substituted with Ala58 and Glu58, which are named 1XQR[A]-5-Ala58 and 1XQR[A]-5-Glu58, respectively.

The clinical data and laboratory findings are gathered for the subjects. The histopathological data and CT scan images of the proband were shown in Figure 3 and also the family information is included in Figure 4a.

A 44-year-old female (III-5, Figure 4a) admitted to the neurology ward with blurred vision and a headache underwent initial evaluation. The patient had a history of weight gain, central obesity, proximal muscle weakness, hypertension (HTN), facial puffiness, and lower extremity edema which had started and progressed 2 years before admission. Due to HTN and mild hypokalemia, the patient was referred to an endocrinologist and admitted and treated with valsartan (80 mg), amlodipine (5 mg), and metoprolol succinate (50 mg). She did not have a familial history of endocrine disorders, but her father suffered from cardiovascular disorder (CVD) and died at the age of 80 due to an ischemic heart attack. Her mother also died at the age of 65, suffering from HTN, uncontrolled diabetes mellitus (DM), and CVD. On admission, she was 84 kg in weight, 163 cm in height, and had a body mass index (BMI) of 31.6 kg/m^2^ and BP = 170/98 mmHg. On neurological assessments, no pathological finding was found in the central nervous system (CNS). Initial lab data assessing resistant HTN showed K+ 3.4 mmol/lit, HCO3- 34.2 mmol/lit, cortisol 8 am 360.2 mcg/L, ACTH 4.2 pg/mL, UFC 364 mcg/24 h, and elevated aldosterone renin ratio (ARR). On an overnight 1 mg dexamethasone suppression test, 8 am cortisol was 25 mcg/dl. Subsequently, a low-dose dexamethasone-suppression (LDDS) test was performed to diagnose Cushing’s syndrome and, on the third day, 8 am serum cortisol was 12.2 mcg/dl (Table S1). Therefore, the patient underwent an abdominal and pelvic CT scan in contrast with the primary diagnosis of Cushing’s syndrome, and the results showed bilateral macronodular enlargement of adrenal glands. The diagnosis of PMAH was made, and a unilateral adrenalectomy was performed on her. Three days after the operation, cortisol at 8 am was 11.01 mcg/dl, and UFC 174 mcg/24h. She lost 10 kg of weight 6 months after surgery and her blood pressure was controlled to be at 130/80 mmHg on valsartan (80 mg) BID. About two years after the surgery, clinical symptoms and lab abnormalities have reappeared and an adrenalectomy of the remaining adrenal gland is currently planned for her. 

The second case was a 46-year-old woman (III-3, Figure 4a), under treatment for hypertension with amlodipine (5 mg) and losartan (50 mg) during the past 2 years before her recent complications. On her current visit to the endocrinologist, she presented with complications such as weight gain (15 kg in 6 months) plus muscle weakness (past 6 months). Three months before her visit, the patient’s sister (III-5) had undergone surgery with the diagnosis of PMAH. Due to her HTN, hypokalemia (3.2 mmol/L), Cushing’s syndrome signs, and familial history, the patient was a candidate for more work-up (assessment shown in Table S1). Further lab work assessing the Cushing’s possibility showed 8 am cortisol level of 340.2 mcg/L, UFC level of 283 mcg/24 h, and ACTH of 1.82 pg/mL. In an overnight dexamethasone suppression test with 1 mg dexamethasone, serum cortisol turned out to be 8.2 mcg/dL. Afterward, the dexamethasone suppression test (LDDS test) confirmed the diagnosis of Cushing’s syndrome, and an abdominal-pelvic CT scan was performed and confirmed PMAH in our patient. Unilateral adrenalectomy was the next step in our patient, which has led to the resolution of any abnormal lab data or clinical manifestation remaining asymptomatic up to the present time.

After a diagnosis of PMAH in her two sisters, a 39-year-old woman (proband III-6, Figure 4a) presented with a weight gain of about 18 kg in 6 months, proximal muscle weakness and facial puffiness, and a plethora of purple stria to her endocrinologist. Considering the mentioned familial history and her clinical signs evaluated for Cushing’s syndrome (initial lab data and further test results are mentioned in Table S1). CT scanning confirmed PMAH as in her two sisters. Eventually, the patient underwent unilateral adrenalectomy leading to complete remission of her signs and symptoms. All three cases’ pathological reports confirmed PMAH findings as well. 

As shown in Table S1, primary aldosteronism was observed in three patients. For the confirmation of primary aldosteronism, a saline infusion test was performed. The infusion test was followed by the intravenous administration of 2 L of isotonic saline over four hours (from 8 a.m. to 12 p.m.), ideally, while the patient is seated. In ordinary people, aldosterone is suppressed, whereas values above 10 ng/dL (277 pmol/L) are consistent with primary aldosteronism [28,29]. An intravenous saline infusion test was conducted in patients’ hospitalization course of admission. After administering 2 L of saline, aldosterone levels for patients were as mentioned: the first case was 15 ng/dl, the second case was 12.5 ng/dl, and the third one was 11 ng/dl. This implies the diagnosis of hyperaldosteronism in these patients. This emphasizes concurrent high levels of cortisol along with aldosterone.

### 3.2. Whole-Exome Sequencing Results

Whole-exome sequencing (WES) was performed to find the genetic variants of III-6 proband related to PMAH and approximately 18 Gb data (121,128,400 million read pairs (150 × 2)) were generated and 115,158,269 read counts were mapped. The quality control of this data exposed 98.1% of the read bases had Q30. Coverage data indicated that 96.9% of the sequenced target regions were at more than 10× reads. Also, as indicated in Table 1, the minimum mean coverage of target regions was 97.7% for >1×, 94.7% for >25×, and 85.4% for >50× of the data. 

### 3.3. Genetic Findings

278,284 variants were detected in the III-6 proband’s annotated VCF file which included 250,964 SNVs and 27,320 indels. Variant filtering, as mentioned above, was performed by the following steps to reduce the number of potentially pathogenic variants. The variants were filtered with the minor allele frequency (MAF) > 0.05 from population databases to identify the rare variants and conserve coding and splicing variants. To evaluate the pathogenicity of rare variants, several in silico analyses were performed such as CADD-PHRED-Score > 15, disease-causing variants evaluated by a Mutation Taster, pathogenic alterations with the DANN-Score (the value range is 0–1), and variants with GERP-Score (−12.3 to 6.17) and Polyphen-2 (greater than 0.9, “probably damaging”). Also, the candidate causative variants were checked in the HGMD and ClinVar databases. Then, we investigated rare OMIM genes, including the PMAH-associated genes. To find variants related to the patient’s phenotypes, we used HPO terms extracted from the HPO database; such as adrenal hyperplasia (HP:0008221), macronodular adrenal hyperplasia (HP:0008231), and Cushing’s syndrome (HP:0003118). 

As a consequence, a novel heterozygous missense variant in the *ARMC5* gene (NM_001105247.2: c.2105C>A; p. Ala702Glu) was identified in proband III-6, which according to ACMG guidelines and Sherloc evidence, classified as “likely pathogenic”. The results of in silico analysis of the pathogenicity of the variants are shown in Table 2. The validation of this variant by Sanger sequencing on proband III-6 was followed by co-segregation analysis in all available family members. The proband III-6 and affected family cases (II-3, III-3, and III-5) have shown this germline variant as a heterozygous state but were absent in unaffected family members (III-7, III-8, and IV-2), except the clinically healthy brother of the proband (III-2). In addition, this heterozygous candidate variant was confirmed by extracted genomic DNA from the tissue of hyperplastic adrenal glands of the proband (Figure 4c). Also, this variant was confirmed in the tissue sample of two sisters (III-3 and III-5) of the proband as a heterozygous state. 

### 3.4. Protein Assessment Results

The predicted secondary structure of “*ARMC5*-Ala702” and “*ARMC5*-Glu702” variants showed that *ARMC5* with high confidence has an alpha–helix structure, regardless of whether it is an “*ARMC5*-Ala702” or “*ARMC5*-Glu702” variant (Figure 4e). The tertiary structure of *ARMC5*, which was adopted from 1XQR[A]-5, represented an increased negative charge in 1XQR[A]-5-Glu58 variant, compared with the 1XQR[A]-5-Ala58 and 1XQR[A]-5 (Figure 4f). Physicochemical evaluation of *ARMC5* variants confirmed that “*ARMC5*-Ala702” is slightly more hydrophobic than “*ARMC5*-Glu702” due to an increased grand average of hydropathy (GRAVY) index. 

## 4. Discussion

Three sisters in the family were diagnosed with primary non-pituitary ACTH-dependent macronodular adrenal hyperplasia. The genetic testing of the proband (III-6) using exome analysis showed a heterozygous germline variant in c.2105C>A on the *ARMC5* gene (NM_001105247.2) in exon 6 which resulted in an amino acid change near BTB/POZ (broad complex/tramtrack/bric-a-brac/pox virus and zinc finger) domain. Overall, 69 mutations were discovered for *ARMC5* based on HGMD data, including missense and nonsense, splicing, and small and gross indels. The detected substitution mutation in this study is a missense variant that changes the wild-type amino acid alanine to glutamic acid at position 702 of the *ARMC5* protein (p. Ala702Glu). Based on HGMD, there are 21 missense mutations that cause macronodular adrenal hyperplasia. 

As predicted by the HOPE project and shown by the protein structure analysis, the mutant residue was found to be bigger and negatively charged and was less hydrophobic than the wild-type residue. Additionally, the wild-type residue has been conserved according to evolutionary methods like GERP (GERP score: 5.0). In addition, *ARMC5* protein showed natural variations in amino acids 702–706 in isoform 1, which had no pathogenic consequences. On the other hand, massive enlargement of adrenal glands was seen in the CT scans of individuals with primary bilateral macronodular adrenal hyperplasia (PBMAH) when deletion mutation occurred in amino acids 702–706 of *ARMC5* [30]. Cancer-related proteins are more charged and less hydrophobic than non-cancerous proteins, according to earlier studies [31,32]. Compared with wild-type *ARMC5*, the charged and less hydrophobic (according to the GRAVY index) replacement of Ala702 by Glu702 increases ARMC5-Glu’s characteristics more in favor of carcinogenic protein (With Ala in position 702). Nevertheless, this conclusion is based on computational thinking and should be empirically confirmed.

Based on ACMG guidelines, the identified missense variant is characterized as a likely pathogenic variant based on the following explanations: this variant is absent in any of the population databases (GenomAD, 1000GP, ExAC, ESP, and Iranome) (PM2); the computational prediction tools support a deleterious effect on the gene *ARMC5* (PP3); co-segregation with the disease in multiple affected family members (PP1) and the patient’s phenotype and family history were highly specific for PMAH with a single genetic etiology (PP4). Thus, PP4 evidence can be considered as a strong piece of evidence by biopsy analysis (pathological tests and results) that is pathognomonic of a specific genetic cause of a disorder and, in the lack of genetic confirmation, would be considered a diagnostic finding [33]. 

To do a comprehensive investigation and increase specificity for the interpretation of this variant, we used the Sherloc classification, which finally predicted this candidate variant as a likely pathogenic variant. These Sherloc points (P) include: (1) This does not exist in any of the population databases (GenomAD, 1000GP, ExAC, ESP, and Iranome) (1P). Multiple general protein predictors (PolyPhen-2, CADD, and DANN) indicate a deleterious effect on *ARMC5* protein (0.5P) as well as weak segregation of the disease with at least three affected individuals (II-3, III-3, and III-5) in this family for dominant genes (1P). This heterozygous variant of the *ARMC5* gene is associated with a dominant disease with well-established diagnostic guidelines and greater than 75% test sensitivity (1P). Also, CT scans and lab findings (pathological results) are pathognomonic for this disorder (1P). We could consider the (c.2105C>A, p. Ala702Glu) variant in *ARMC5* as a causative variant for PMAH; nevertheless, to discover other affected unrelated families, further studies on the effects of this variant on protein structure and functional studies would help gather more evidence.

The *ARMC5* gene was found to be the major genetic cause of PMAH and included nearly 80% of familial cases with PMAH and 30% of apparently sporadic forms [4,12]. *ARMC5* has germline and somatic mutations, according to genetic analysis [7]. The germline-detected variant was investigated in blood samples of family members which were segregated into affected members but not unaffected ones. PMAH with *ARMC5* mutations has an autosomal dominant inheritance pattern with incomplete penetrance [34]. Stratakis et al. also suggested that *ARMC5* inactivation in mice is age-dependent and plays the role of *ARMC5* haploinsufficiency on adrenocortical function [35]. This article mentioned *ARMC5* heterozygote mice (Armc5+/−) at 12 months of age replicates the genetics of younger patients with *ARMC5*-inactivating mutations in which their corticosterone levels decreased. However, around 18 months of age, these corticosterone levels were compensated for and increased, indicating a role for the PKA and Wnt/ β catenin signaling pathways in the age-dependent development of PMAH in older adults with *ARMC5* mutations.

In addition, the manifestation of disease could be observed in late-onset age (the fifth and sixth decades of life) [36,37,38]. For example, a PMAH patient with the mutation was detected after the sixth decade of life [34] and several AIMAH patients with a mean age of 56 years showed clinical symptoms [36]. The putative germline mutation was confirmed and segregated into three cases (III-3, III-5, and III-6) that were diagnosed with PMAH based on clinical and laboratory findings in this family lineage. However, we discovered the proband’s brother, an asymptomatic *ARMC5* variant carrier (III:2), among the family members. This 39-year-old unaffected member exhibited no laboratory nor clinical PMAH symptoms. Therefore, this young asymptomatic male carrier with a germline *ARMC5* mutation might express the disease in the following decades of his life, taking into account that the disease may display incomplete penetrance due to the potential of the diagnosis at late-onset age.

PMAH patients with *ARMC5* mutations have a higher number of nodules, larger nodules, and autonomous cortisol secretion with low circulating ACTH than cases without *ARMC5* mutations [30,39,40]. *ARMC5*, which contains an N-terminal armadillo repeat domain and a C-terminal BTB, has a role in the regulation of apoptosis and steroidogenesis. Functional studies showed that the inactivation of *ARMC5* leads to the inhibition of apoptosis and decreases the expression of MC2R and steroidogenic enzymes which may be involved in adrenal hyperplasia. It seems that hypercortisolism in PMAH is related to the increased number of adrenocortical cells supposedly caused by mutations in *ARMC5* and the contribution of aberrant G-coupled receptors and intra-adrenal ACTH in hyperplastic adrenal cells is suggested by mechanisms of the autonomous cortisol production in PMAH [34,41,42].

As mentioned above, the germline and somatic variants were described in *ARMC5* and the germline variants were documented in sporadic and familial PMAH cases in 50% of investigated individuals [4,34,42]. *ARMC5* was considered as a tumor suppressor gene and the germline variants in *ARMC5* should be transmitted to family members in pedigree. The second somatic variants or another second hit may induce tumorigenesis and/or contribute to the progression of macronodules [4,42,43,44]. Hence, the study has some limitations. Due to a lack of sufficient funding, we were unable to investigate the functional consensuses of the detected variant and to sequence the whole *ARMC5* gene to find any somatic variations. Given that somatic mutations can only be detected by Sanger sequencing in around 20% [45] and that many genes are involved in the pathogenesis of PMAH, NGS techniques must be used for all patient tissues. These techniques thus include all potential pathogenic genes and provide sufficient coverage to find low-frequency somatic mutations.

In conclusion, our results indicate that a novel missense variant, c.2105C>A, in the *ARMC5* gene might be the genetic cause of PMAH, and based on the standard guidelines, the recognized variant was characterized as likely pathogenic; however, we essentially advise performing functional analysis to assess the distinct role of the variant pathogenicity. The finding of pathogenic/likely pathogenic *ARMC5* gene mutations enhances the early stage of PMAH diagnosis (in an age-dependent way). Consequently, the confirmation of a germline variation is used to conduct healthy life-long follow-ups in asymptomatic variant carriers of PMAH families. Furthermore, the diagnosis of an inherited germline mutation in cancer predisposition genes, such as *ARMC5*, can be applied in preimplantation genetic diagnosis (PGD) [46,47,48] to prevent the occurrence of cancerous patients.

## Figures and Tables

**Table 1:** The summary explanation of data analysis from the WES.

Analytical Characteristic	Proband III-6
Total number of reads	121,128,400
Average read length (bp)	150
Target region (Mbp)	36
% Bases QV > 30	98.16
% Initial mappable reads	99
% Minimum coverage of target regions (For depth 1×, 5×, 10×, 25×, 50× and 100×)	97.7, 97.3, 96.9, 94.7, 85.4 and 52.7
% Of duplicate reads (pre-alignment)	25
% Of duplicate reads (post-alignment)	6
% On target reads (post-alignment)	55 66,811,296 (reads)

**Table 2:** The variant illustration of recognized variants such as in silico analysis and variant frequency in population databases.

Patient	Proband III-6
Variant Definition	
-Gene name	*ARMC5* (NM_001105247.2)
-Varian name	c.2105C>A
-Protein change	p. Ala702Glu
-Chromosome position (GRCh37)	Chr16: 31477507
-Zygosity	Heterozygote
In-silico predictive tools	
-CADD (Phred score)	25 (deleterious)
-DANN	0.9948 (deleterious)
-GERP	5
-Mutation taster	Disease-causing
-Polyphen	Probably-damaging
Population databases	
-1000 GP	-
-ExAC	-
-ESP	-
-GnomAD	-
-Iranome	-
Related phenotypes (OMIM number)	ACTH-independent macronodular adrenal hyperplasia 2/AIMAH2 (OMIM: 615954)
Variant classification (Evidence based on ACMG guideline) (Evidence based on Sherloc)	Likely pathogenic (PM2, PP3, PP4-strong, and PP1) (PM2, PP3, PP4, PP1, and LAB-assay points)

## Data Availability

Not applicable.

## Supplementary Materials

The following supporting information can be downloaded at: https://www.mdpi.com/article/10.3390/diagnostics12123028/s1, Table S1: The Laboratory data of the Subjects.

## Abbreviations

PMAH	Primary macronodular adrenocortical hyperplasia
WES	Whole-exome sequencing
AIMAH	ACTH-independent macronodular adrenal hyperplasia
CS	Cushing’s syndrome
LOH	Loss of heterozygosity
SNP	Single-nucleotide polymorphism
*ARMC5*	Armadillo repeat-containing protein 5
*APC*	Adenomatous polyposis coli gene
*GNAS*	Guanine nucleotide-binding protein alpha-stimulating activity polypeptide
*PRKACA*	cAMP-dependent protein kinase catalytic subunit alpha
*PDE11A*	phosphodiesterase 11A
*PDE8B*	phosphodiesterase 8B
SAM	Sequence Alignment/Map
SNVs	Single nucleotide variants
IGV	Integrated genome viewer
VCF	Variant call format
Indels	Insertions or deletions
LOF	Loss-of-function
HGMD	Human Gene Mutation Database
ESP	Exon Sequencing Projects
CADD	Combined Annotation Dependent Depletion
HPO	Human Phenotype Ontology
OMIM	Online Mendelian Inheritance in Man
ACMG	American College of Medical Genetics and Genomics guideline
Sherloc	Semiquantitative, hierarchical evidence-based rules for locus interpretation
SMR	SWISS-MODEL Repository
CVD	Cardiovascular disorder
HTN	Hypertension
DM	Diabetes mellitus
CNS	Central nervous system
BMI	Body mass index
ARR	Aldosterone renin ratio
LDDS	Low-Dose Dexamethasone-Suppression
MAF	Minor allele frequency
PGD	Preimplantation genetic diagnosis
