# Supplementary material for: A Novel ARMC5 Germline Variant in Primary Macronodular Adrenal Hyperplasia Using Whole-Exome Sequencing
Source: Diagnostics (Basel). 2022 Dec 2;12(12):3028. doi: 10.3390/diagnostics12123028 (PMC9777150; doi:10.3390/diagnostics12123028)
Supplement: Supplementary file 1 [file diagnostics-12-03028-s001.zip › diagnostics-2025537-supplementary.pdf]

Table S1. The Laboratory data of the Subjects. ALT, alanine transaminase; AST, aspartate aminotransferase; FPG, fasting plasma glucose; TSH, thyroid-stimulating hormone; eGFR, estimated glomerular filtration rate; ARR, Aldosterone-to-renin ratio; ACTH, adrenocorticotropic hormone; LDDST, Dexamethasone suppression test.

Subjects	Test	Result	Reference (unit)
**Case 1 (III-5)**	WBC	8.03	4–10 (*109/L)
	Hemoglobulin	128	120–160 (g/L)
	Platelet	141	100–300 (*109/L)
	ALT	21	4–41 (U/L)
	AST	15	4–40 (U/L)
	Creatinine	85	59–104 (μmol/L)
	Uric acid	453.4	202.3–416.5 (μmol/L
	eGFR	91	> 90 (ml/min/1.73 m2)
	HCO3−	33.3–33.8 ↑	22–29 (mmol/L)
	K+	2.71–3.73 (low)	3.50–5.10 (mmol/L)
	FPG 6.09 3.90–6.10 (mmol/L)	109	70-100 (mg/dl)
	HbA1c	7.3% ↑	4.40–6.50%
	TSH	0.71	0-5-4.5
	Renin	0.77	0.10–6.56 (ng/ml/hour)
	Aldosterone	23.75	7–30 (ng/dl)
	ARR	30.84 ↑	< 25
	Aldosterone after Saline test	15	<5 (ng/dl)
	Adrenaline	1.86	1.21–2.30 (nmol/L)
	Noradrenaline	2.20	1.31–2.51 (nmol/L)
	ACTH	4.23 (low)	1.60–13.9 (pmol/L)
	Cortisol (8AM)	360.20 ↑	62–194 (μg/L)
	Cortisol (4PM)	245.20 ↑	23–119 (μg/L)
	Free cortisol in 24 h urine	364.24↑	36–137 (μg/24 h)
	LDDST: cortisol (8AM)	12,2 ↑	< 1.8 (μg/dL)
**Case 2 (III-3)**	WBC	5.03	4–10 (*109/L)
	Hemaglobulin	118	120–160 (g/L)
	Platelet	254	100–300 (*109/L)
	ALT	40	4–41 (U/L)
	AST	35	4–40 (U/L)
	Creatinine	100	59–104 (μmol/L)
	Uric acid	345.4	202.3–416.5 (μmol/L
	eGFR	90	> 90 (ml/min/1.73 m2)
	HCO3−	24.3–29.8 ↑	22–29 (mmol/L)
	K+	3.40–3.90 (low)	3.50–5.10 (mmol/L)
	FPG 6.09 3.90–6.10 (mmol/L)	99	70-100 (mg/dl)
	HbA1c	5.39% ↑	4.40–6.50%
	TSH	1.00	0-5-4.5
	Renin	0.75	0.10–6.56 (ng/ml/hour)
	Aldosterone	26.75	7–30 (ng/dl)
	ARR	31.84 ↑	< 25
	Aldosterone after Saline test	12.5	<5 (ng/dl)
	Adrenaline	2.06	1.21–2.30 (nmol/L)
	Noradrenaline	1.76	1.31–2.51 (nmol/L)
	ACTH	1.82	1.60–13.9 (pmol/L)
	Cortisol (8AM)	340.2	62–194 (μg/L)
	Cortisol (4PM)	241.20 ↑	23–119 (μg/L)
	Free cortisol in 24 h urine	316 ↑	36–137 (μg/24 h)
	LDDST: cortisol (8AM)	17.5 ↑	< 1.8 (μg/dL)
**Case 3 (III-6)**	WBC	6.03	4–10 (*109/L)
	Hemoglobin	136	120–140 (g/L)
	Platelet	223	100–300 (*109/L)
	ALT	10	4–41 (U/L)
	AST	14	4–40 (U/L)
	Creatinine	55	59–104 (μmol/L)
	Uric acid	253.4	202.3–416.5 (μmol/L
	eGFR	91	> 90 (ml/min/1.73 m2)
	HCO3−	28.3–29.8 ↑	22–29 (mmol/L)
	K+	3.51–3.73 (low)	3.50–5.10 (mmol/L)
	FPG 6.09 3.90–6.10 (mmol/L)	110	70-100 (mg/dl)
	HbA1c	6.1% ↑	4.40–6.50%
	TSH	1.09	0-5-4.5
	Renin	0.34	0.10–6.56 (ng/ml/hour)
	Aldosterone	35.89	7–30 (ng/dl)
	ARR	32.84 ↑	< 25
	Aldosterone after Saline test	11	<5 (ng/dl)
	Adrenaline	2.06	1.21–2.30 (nmol/L)
	Noradrenaline	1.40	1.31–2.51 (nmol/L)
	ACTH	2.63 (low)	1.60–13.9 (pmol/L)
	Cortisol (8AM)	432.20 ↑	62–194 (μg/L)
	Cortisol (4PM)	245.20 ↑	23–119 (μg/L)
	Free cortisol in 24 h urine	388.24↑	36–137 (μg/24 h)
	LDDST: cortisol (8AM)	14.5 ↑	< 1.8 (μg/dL)
